# Supplementary material for: Simple, reference-independent assessment to empirically guide correction and polishing of hybrid microbial community metagenomic assembly
Source: PeerJ. 2024 Nov 8;12:e18132. doi: 10.7717/peerj.18132 (PMC11552494; doi:10.7717/peerj.18132)
Supplement: Supplemental Information 3 — Rationale/Advantages in this used to support or justify application in this study over others were almost entirely arbitrary or based on subjective criteria; better-performing tools may exist for the same purpose but were not used for this study. Citations were only provided here for demonstration of specific attributes related to the rationale beyond the tool’s release publication. [file peerj-12-18132-s003.docx]

**Table S1.** Tool choice, its purpose in this manuscript, and the rationale for or advantages of its use here. Rationale/Advantages in this used to support or justify application in this study over others were almost entirely arbitrary or based on subjective criteria; better-performing tools may exist for the same purpose but were not used for this study. Citations were only provided here for demonstration of specific attributes related to the rationale beyond the tool’s release publication.

| **Tool** | **Purpose** | **Rationale/Advantages** |
| --- | --- | --- |
| Illumina MiSeq  (Nextera XT kit) | Short read sequencing | Prevalence in field, in-house capability |
| Oxford Nanopore Technologies MinION  (R9.4.1 flowcell) | Long read sequencing | Prevalence in field, in-house capability |
| Guppy^78^  (v 4.0.11, dna_r9.4.1_450bps_hac) | Long read base-calling | Prevalence in field, in-house capability standard |
| BBduk^80^  (BBtools v 37.76) | Short read QC | One-step trimming and filtering, companion tools |
| porechop^81^  (v 0.2.3_seqan2.1.1) | Long read QC | Prevalence in field |
| hybridSPAdes^20^  metaSPAdes^18^  (v 3.15.4) | Short read, long read, and short read first hybrid assembly | Prevalence in field, other tools use it therefore reducing technical variation |
| OPERA-MS^27^  (v 0.9.0) | Hybrid assembly | Prevalence in field, SPAdes-dependent |
| Unicycler^82^  (v 0.4.9b) | Short read, long read, and short read first hybrid assembly | Developed for hybrid assembly to recover high-quality and circular microbial genomes, SPAdes-dependent short read assemblies, long read hybrid assembly using Racon |
| Canu^21^  (v 1.8) | Long read assembly | Prevalence in field, often compared (or used in comparative studies), metagenome-optimization possible |
| Flye^14^  (v 2.9-b1768) | Long read assembly | Prevalence in field, often compared (or used in comparative studies), metagenome-optimization integrated |
| Trycycler^8^  (v 0.4.1) | Long read subsampling | Ease of use |
| Racon^9^  (v 1.3.1) | Long read correction | Prevalence in field, often compared (or used in comparative studies), minimal computational requirements, other tools use it therefore reducing technical variation |
| Pilon^13^  (v 1.23) | Short read polishing | Prevalence in field, often compared (or used in comparative studies), minimal computational requirements, data rich logging, companion tools |
| Minimap2^91^  (v 2.17-r941) | Long read recruit for correction | Prevalence in field, ease of use with Racon |
| BBmap^80^  (BBtools v 37.76) | Short read recruitment for polishing | One-step mapping and filtration, companion tools |
| metQuast^69^  (v 5.0.2) | General assembly characteristics | Prevalence in field, ease of use |
| RNA polymerase subunit B (*rpoB*) gene  (pfam^93^ PF04563.15, downloaded 2020-06-09) | Beta diversity | Universal to all three domains of life in essentially single copy, use in field, previous application to the system |
| readcoverage2  (mmlong^92^ v 0.1.2) | Read depth profiling | Ease of integration with binner, calculated long read depth properly |
| Non-Metric multi-Dimensional Scaling (NMDS)  (vegan^104^ v 2.5-7,  R^105^ v 4.1.2) | Beta diversity | Use in (related) field(s) |
| Metabat2^17^  (v2.12.1) | Automated binning | Prevalence in field, often compared (or used in comparative studies), low computational requirements |
| GTDB-tk^72^  (v 1.6.0, db v r202) | Microbial taxonomy inference | Prevalence in field, little competition |
| BUSCO^101^  (v 5.1.2) | Marker gene identification | Used in field but can be extended more broadly, assesses gene fragmentation |
| CheckM^71^  (v 1.1.3) | Marker gene identification and microbial genome completion estimation | Prevalence in field, assesses gene redundancy |
| Prodigal^102^  (v 2.6.3) | Gene calling for several analyses | Prevalence in field, other tools use it therefore reducing technical variation and reducing computational costs |
| IDEEL^84^  (downloaded 2023-05-13) | Gene fragmentation | Use in field, ease of use, little competition |
| BBmap^80^  (BBtools v 37.76) | Short read recruitment analysis | One-step mapping and filtration, data rich output summaries |
| ALE^103^  (downloaded 2024) | Assembly quality estimation form short read recruitment profiles | Used in microbial genome assembly comparative studies |
