# Supplementary material for: Simple, reference-independent assessment to empirically guide correction and polishing of hybrid microbial community metagenomic assembly
Source: PeerJ. 2024 Nov 8;12:e18132. doi: 10.7717/peerj.18132 (PMC11552494; doi:10.7717/peerj.18132)
Supplement: Supplemental Information 5 — Paired read counts are shown for MiSeq datasets. OLR, oxygen-limited bioreactor; NLR, nitrogen-limited bioreactor; MiSeq, Illumina MiSeq platform; ONT, Oxford Nanopore Technologies platform. [file peerj-12-18132-s005.docx]

**Table S2.** Sequencing yields for both reactors and sequencing platforms after quality control and trimming. OLR, oxygen-limited bioreactor; NLR, nitrogen-limited bioreactor; MiSeq, Illumina MiSeq platform; ONT, Oxford Nanopore Technologies platform.

| **Reactor** | **Sequencer** | **Read count*** | **Read bps**  **(total)** | **Read length (mean)** |
| --- | --- | --- | --- | --- |
| OLR | MiSeq | 7,568,591 | 2,031,927,441 | 268 |
| OLR | ONT | 318,070 | 3,128,778,886 | 9,837 |
| NLR | MiSeq | 8,733,498 | 2,300,830,797 | 263 |
| NLR | ONT | 416,527 | 4,014,258,886 | 9,638 |

* Paired read counts and bps for Illumina data, *i.e.*, total R1 and R2 reads and bps divided by 2.
