# Supplementary figures and images for: Simple, reference-independent assessment to empirically guide correction and polishing of hybrid microbial community metagenomic assembly
Source: PeerJ. 2024 Nov 8;12:e18132. doi: 10.7717/peerj.18132 (PMC11552494; doi:10.7717/peerj.18132)

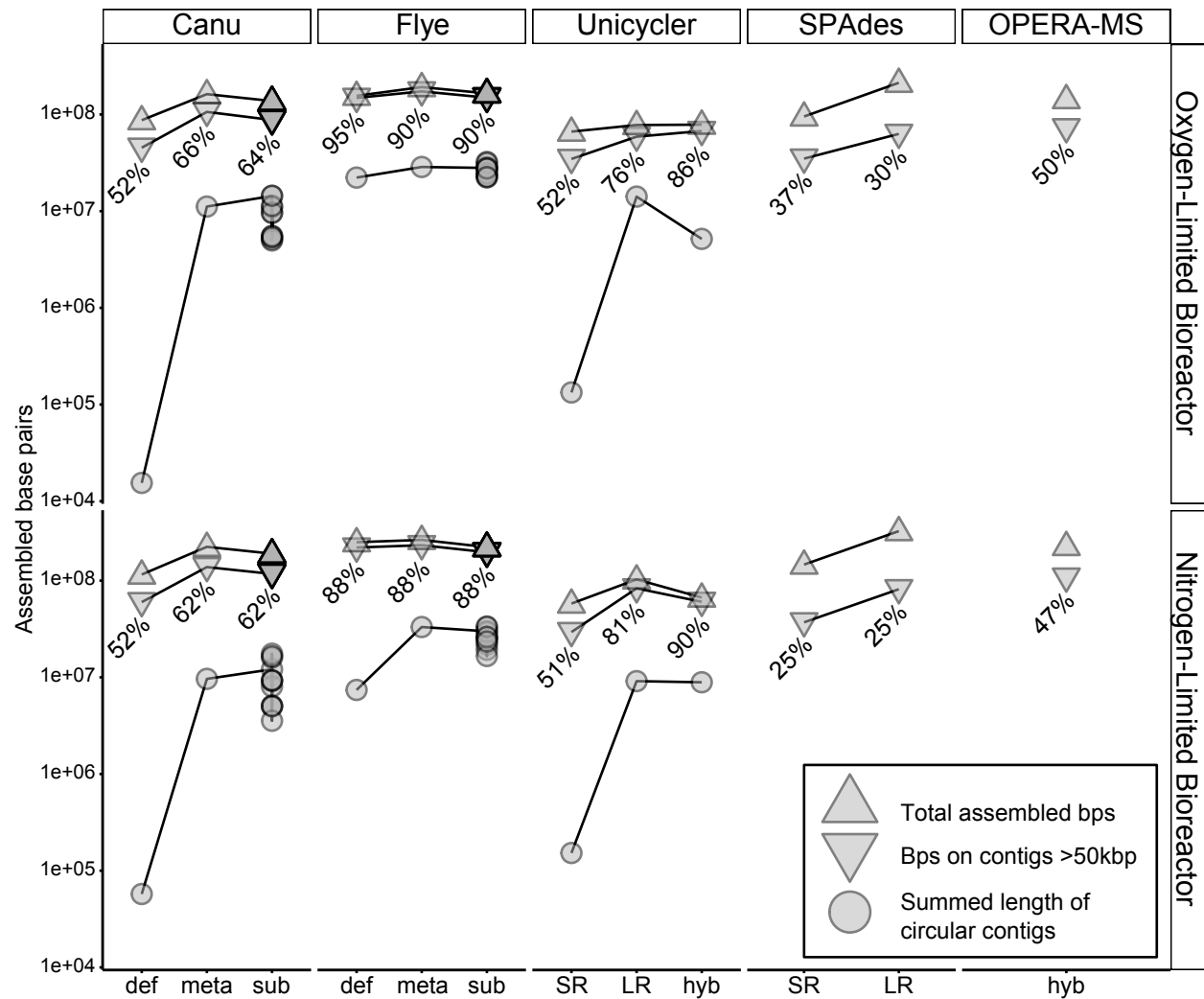

Supplement: Supplemental Information 6 — In each panel, separated vertically by reactor and horizontally by assembler, the total assembled bps (upward-pointing triangles), bps on contigs >50 Kbp (downward pointing triangles), and bps in circular contigs (circles) are shown on the y-axis and grouped by the assembly type or setting on the x-axis. Numbers indicate the percentage of total bps on the larger ( >50 Kbps) contigs. Abbreviations are as follows: def, default settings; meta, metagenome-optimized settings; sub, assembly of sub-sampled reads; SR, short read only metagenomic assembly; LR, long read only assembly; hyb, hybrid assembly. [file peerj-12-18132-s006.pdf]

**A**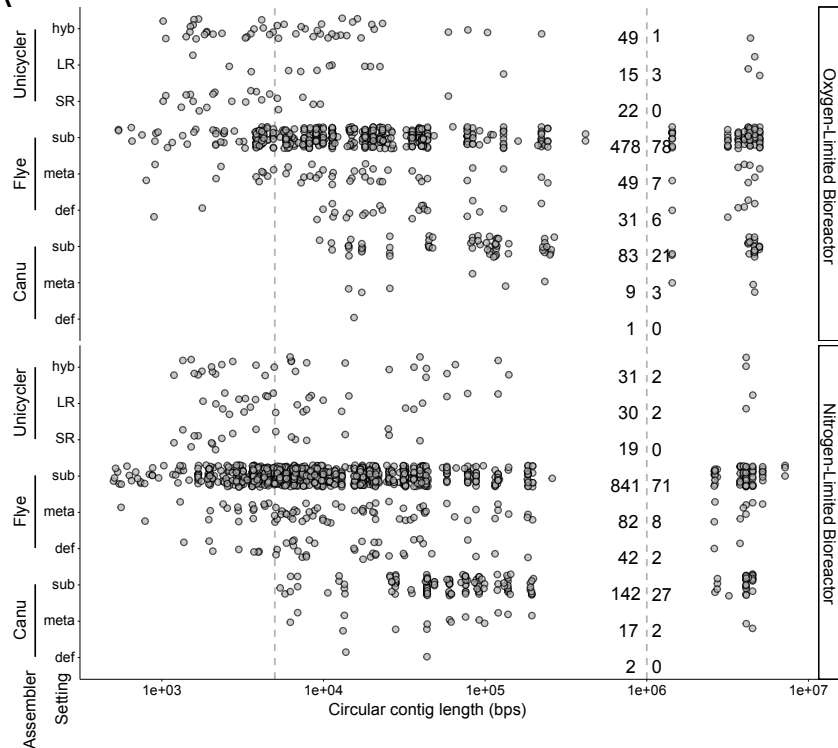**B**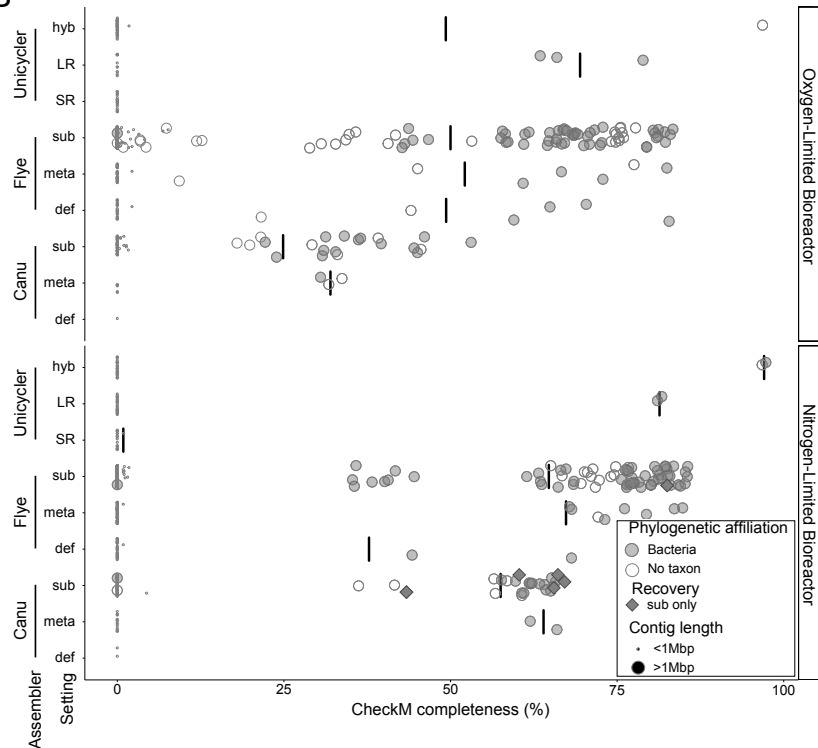

Supplement: Supplemental Information 7 — The two bioreactors are separated over the vertical panels. (A) Length distribution of circular contigs for each assembler. Vertical dashed lines indicate common thresholds for considering complete genomes or chromosomes, >5 Kbp for plasmids and phages, and >1 Mbp for microbial genomes, though exceptions exist. The number of contigs passing these thresholds are indicated next to the right-most vertical line. (B) Completion of circular contigs estimated using lineage-specific phylogenetic markers. Filled circles indicate a circular contig with bacterial phylogenetic markers, empty circles indicate a circular contig lacking sufficient data to assign to the bacterial domain, and filled diamonds indicate a bacterial lineage only circularized in sub-sampled assemblies. Black horizontal bar represents the mean of circular contigs with non-zero completion estimates. Abbreviations are as follows: def, default settings; meta, metagenome-optimized settings; sub, assembly of sub-sampled reads; SR, short read only metagenomic assembly; LR, long read only assembly; hyb, hybrid assembly. [file peerj-12-18132-s007.pdf]

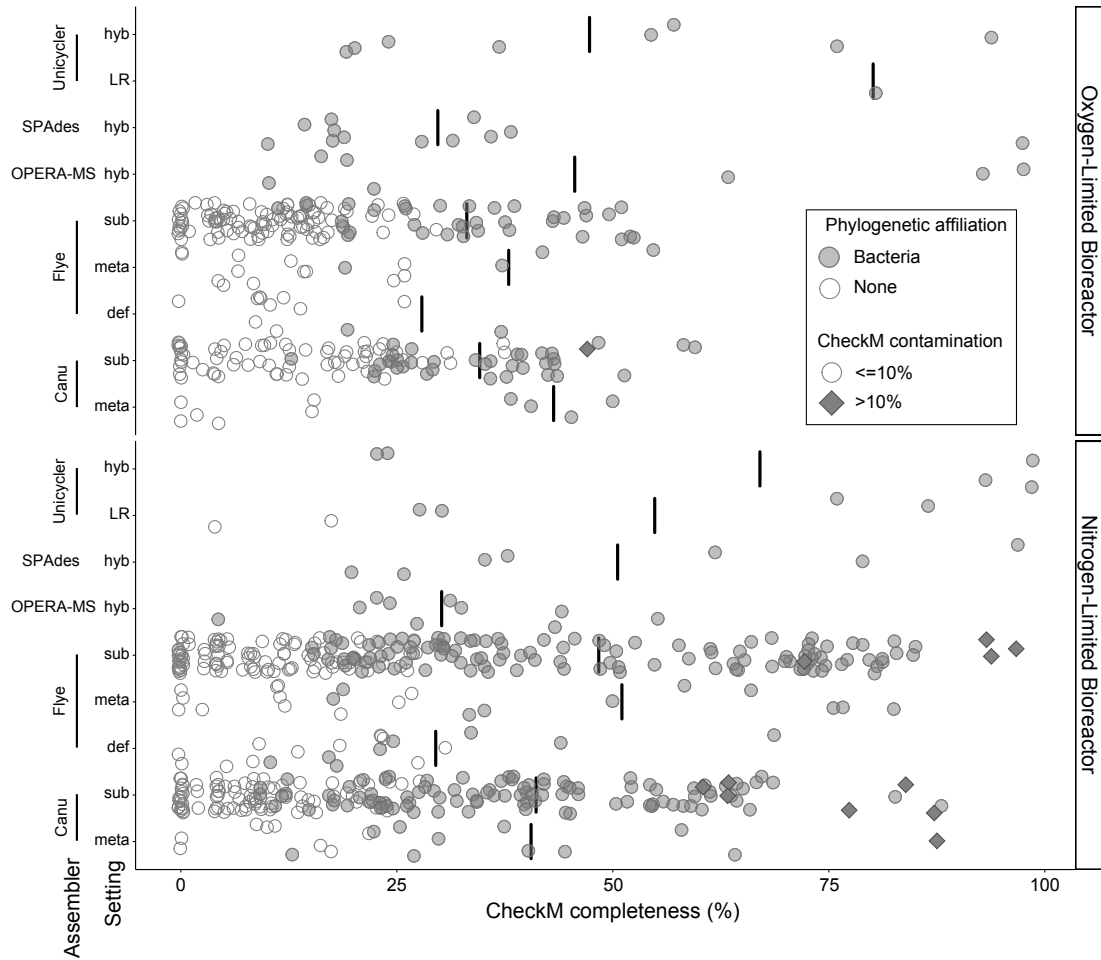

Supplement: Supplemental Information 8 — The two bioreactors are separated over the vertical panels. Filled circles indicate >1 Mbp contigs containing bacterial phylogenetic markers, empty circles indicate >1 Mbp contigs lacking sufficient data to assign to the bacterial domain, and filled diamonds indicate a contig with >10% redundancy of bacterial phylogenetic markers. Black horizontal bars represent the mean of >1 Mbp contigs with sufficient data to be affiliated with the bacterial domain. Abbreviations are as follows: def, default settings; meta, metagenome-optimized settings; sub, assembly of sub-sampled reads; SR, short read only metagenomic assembly; LR, long read only assembly; hyb, hybrid assembly. [file peerj-12-18132-s008.pdf]

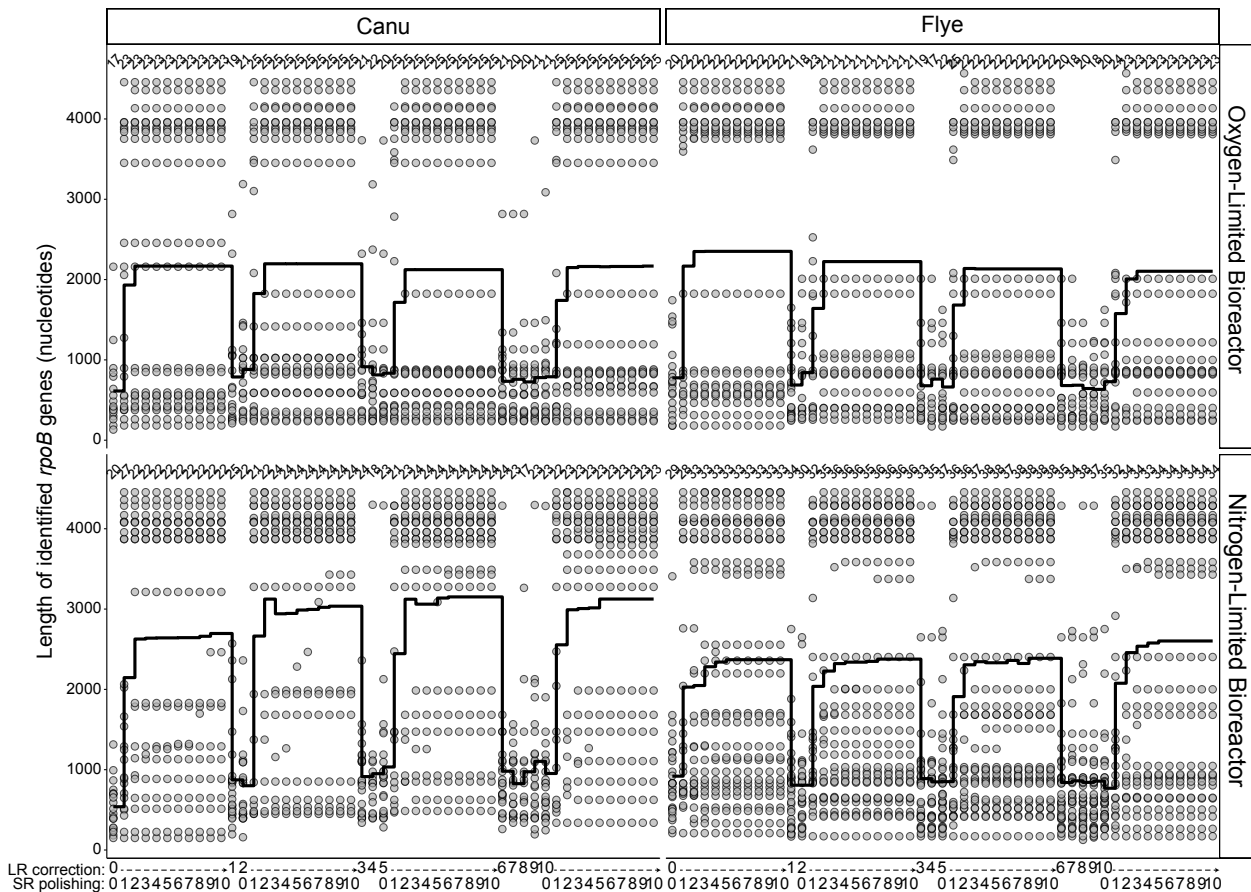

Supplement: Supplemental Information 9 — The two bioreactors are separated over vertical panels, the two LR assemblers over the horizontal panels. The LR correction and SR polishing iterations are spread across the x-axis so that the ten SR polishing steps are immediately to the right of the preceding LR correction step. Points are the length of each rpoB gene in the assembly, with the black line showing the mean length for the assembly and the text above the points indicating the count. [file peerj-12-18132-s009.pdf]

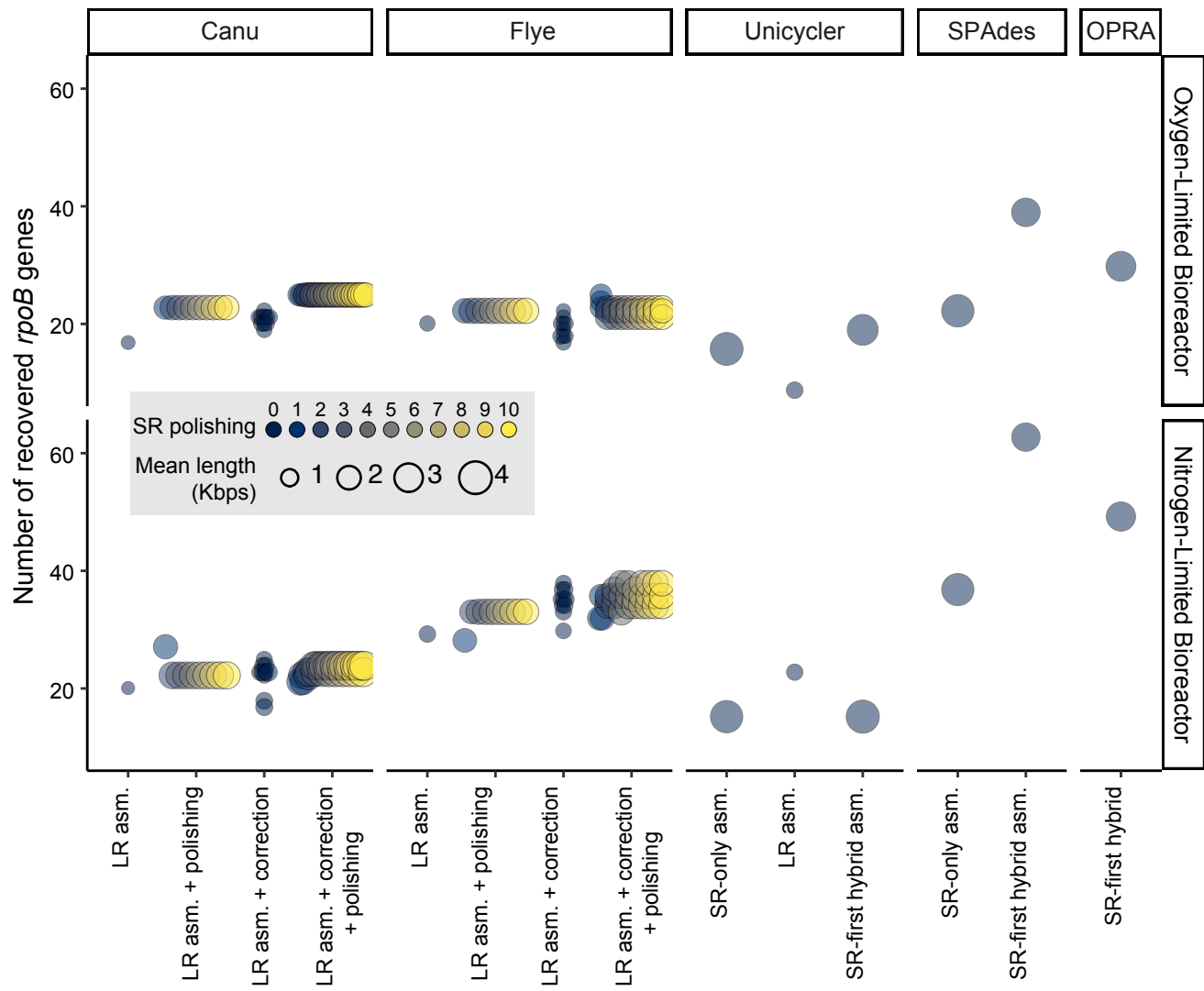

Supplement: Supplemental Information 10 — The two bioreactors are separated over vertical panels, the assemblers over the horizontal panels. Colored points represent one assembly, sized by gene length, and are grouped along the x-axis by the type of reads used for assembly and post-assembly processing. [file peerj-12-18132-s010.pdf]

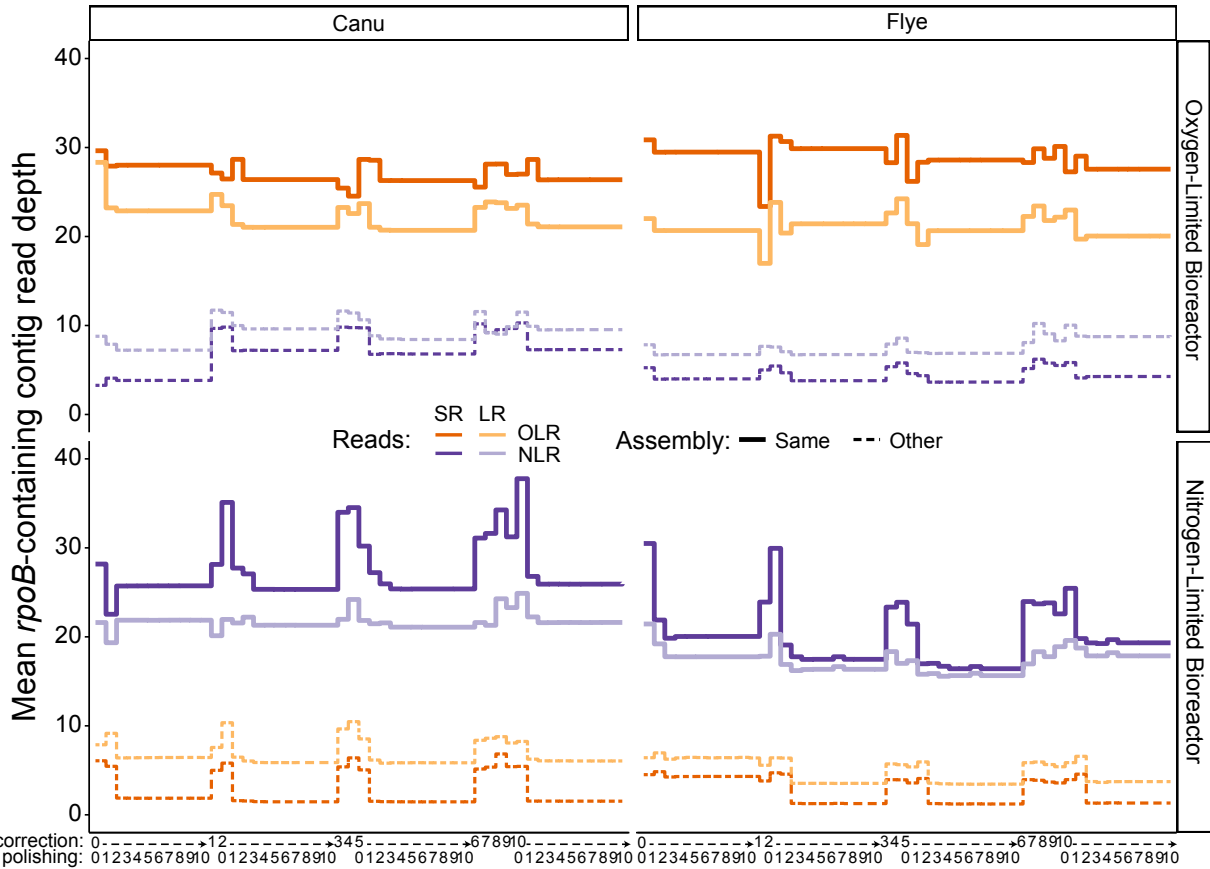

Supplement: Supplemental Information 11 — The two bioreactors are separated over vertical panels, the two LR assemblers over the horizontal panels. The LR correction and SR polishing iterations are spread across the x-axis so that the ten SR polishing steps are immediately to the right of the preceding LR correction step. Colored lines indicate the read source used for mean read depth calculation. [file peerj-12-18132-s011.pdf]

Number of medium quality or better automated bins

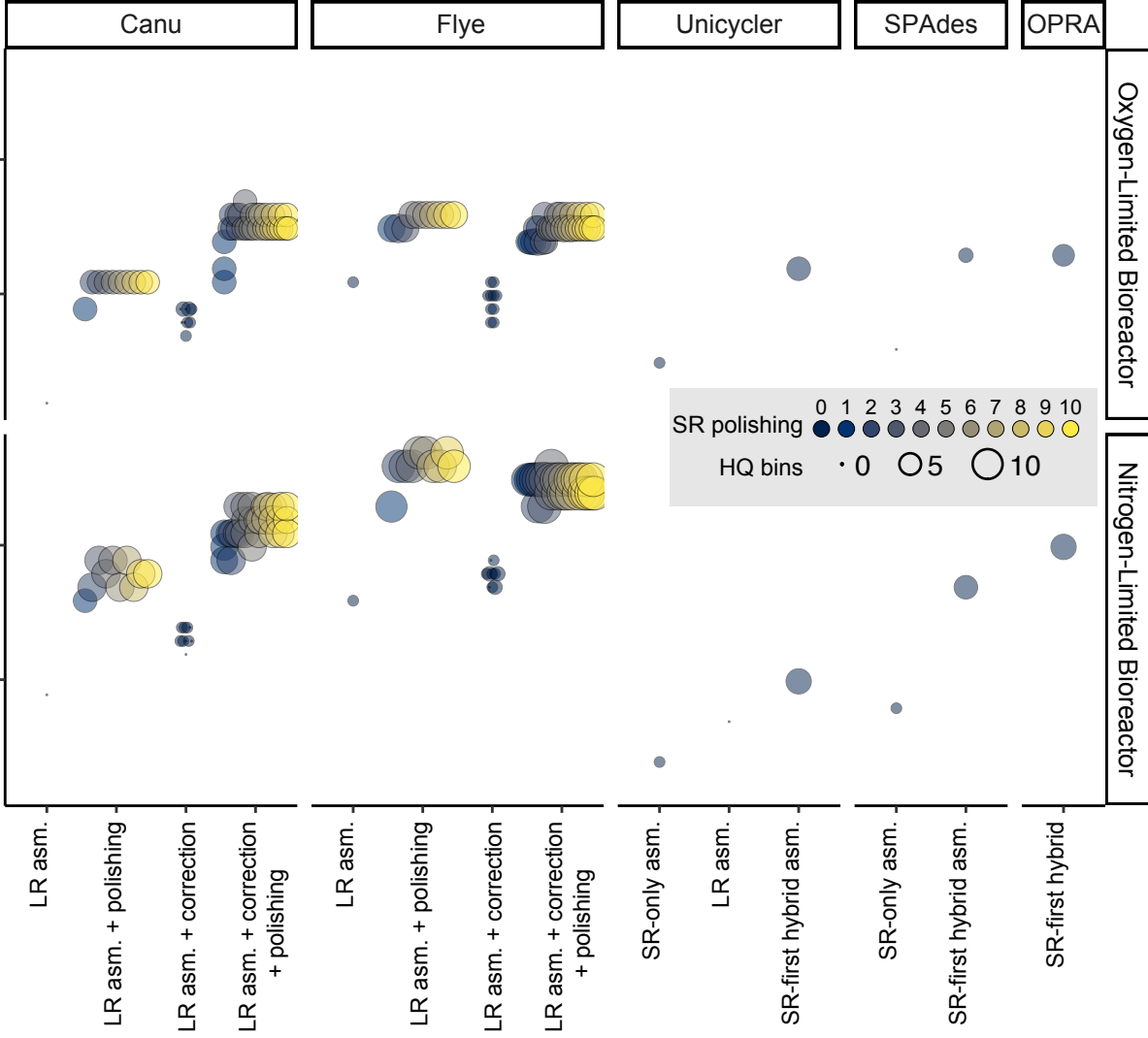

Supplement: Supplemental Information 13 — The two bioreactors are separated over vertical panels, the assemblers over the horizontal panels. Colored points represent one assembly, sized by the number of High-Quality (HQ) bins, and are grouped along the x-axis by the type of reads used for assembly and post-assembly processing. [file peerj-12-18132-s013.pdf]

Proportion of max observed value  
(per data subset, bioreactor, and assembler combination)

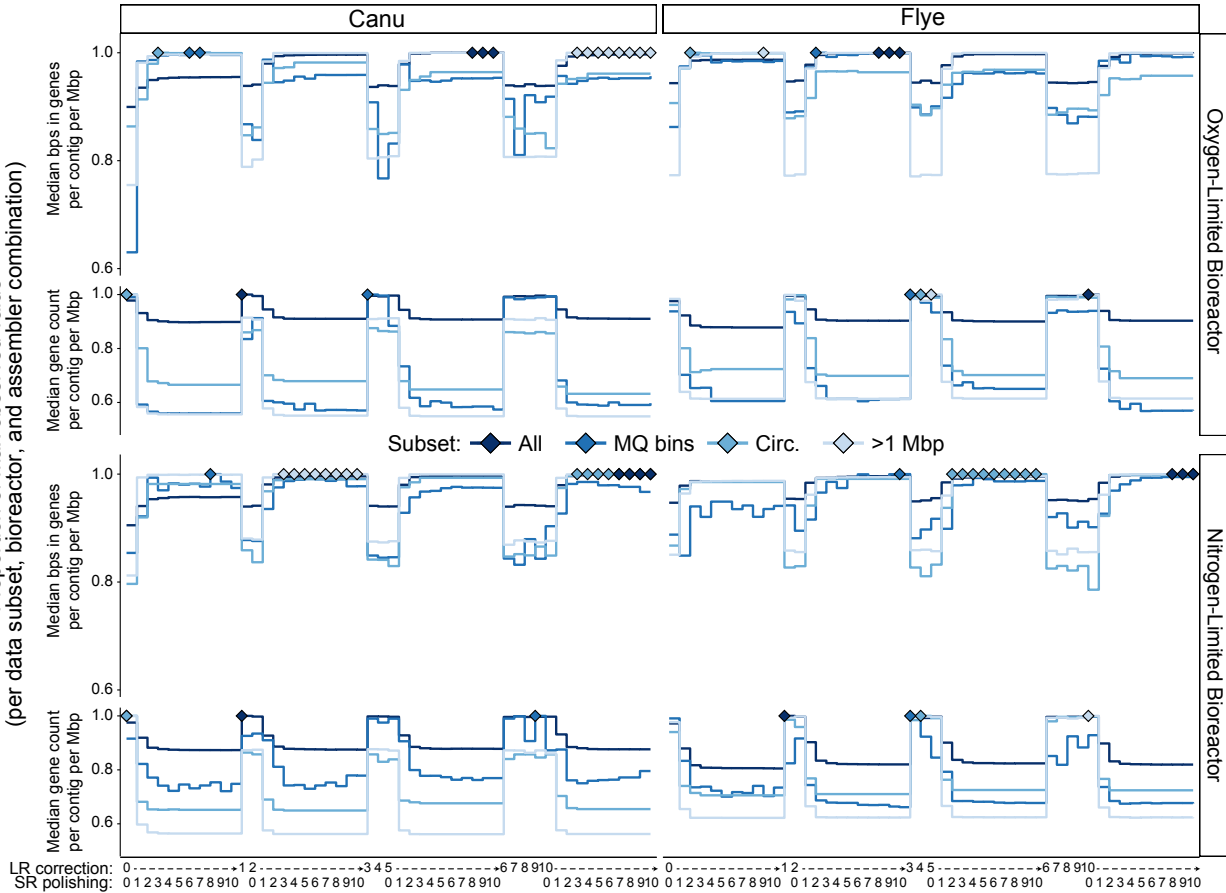

Supplement: Supplemental Information 15 — The two bioreactors are separated over vertical panels, the two LR assemblers over the horizontal panels. The LR correction and SR polishing iterations are spread across the x-axis so that the ten SR polishing steps are immediately to the right of the preceding 0, 2, 5, or 10 LR correction step. Colored lines show the proportion of the maximum value for fractions of the assemblies throughout the LR correction and SR polishing iterations –the entire assembly (All), medium quality or better bins (MQ bins), circular contigs >10 Kbp (Circ.), and long contigs that may be complete but not circular bacterial genomes ( >1 Mbp). Diamonds highlight the stages at which the maximum value occurred. [file peerj-12-18132-s015.pdf]

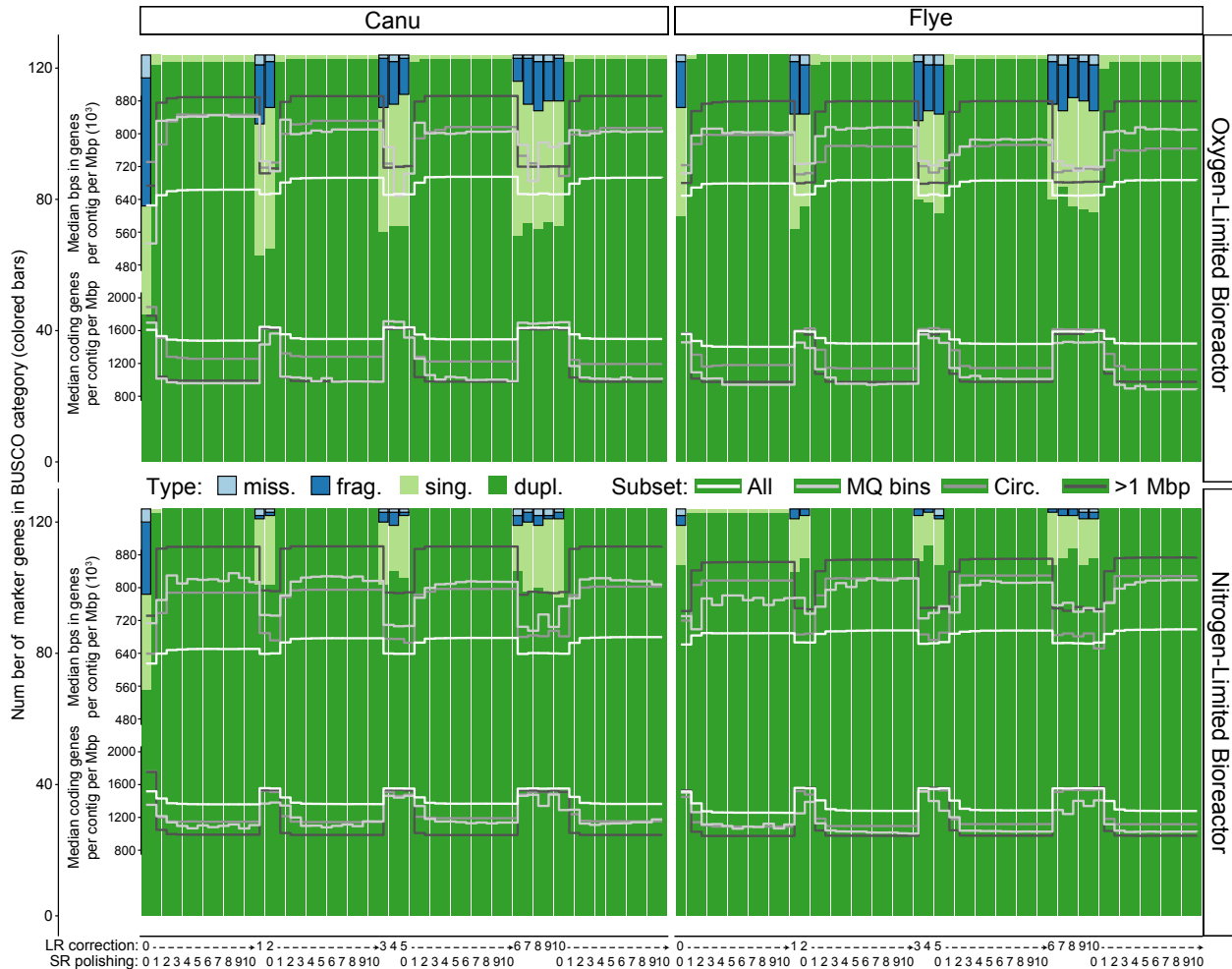

Supplement: Supplemental Information 16 — The two bioreactors are separated over vertical panels, the two LR assemblers over the horizontal panels. The LR correction and SR polishing iterations are spread across the x-axis so that the ten SR polishing steps are immediately to the right of the preceding 0, 2, 5, or 10 LR correction step. Colored bars show the number of bacterial marker genes in each BUSCO category –missing (miss.), fragmented (frag.), complete and single copy (sing.), complete and duplicated (dupl.) –for the entire assembly, and the gray lines indicate the median number of genes or bps in genes per contig per Mbp scaled to overlay the bars for several fractions of the assemblies throughout the LR correction and SR polishing iterations –the entire assembly (All), medium-quality or better bins (MQ bins), circular contigs >10 Kbp (Circ.), and long contigs that may be complete but not circular bacterial genomes ( >1 Mbp). [file peerj-12-18132-s016.pdf]

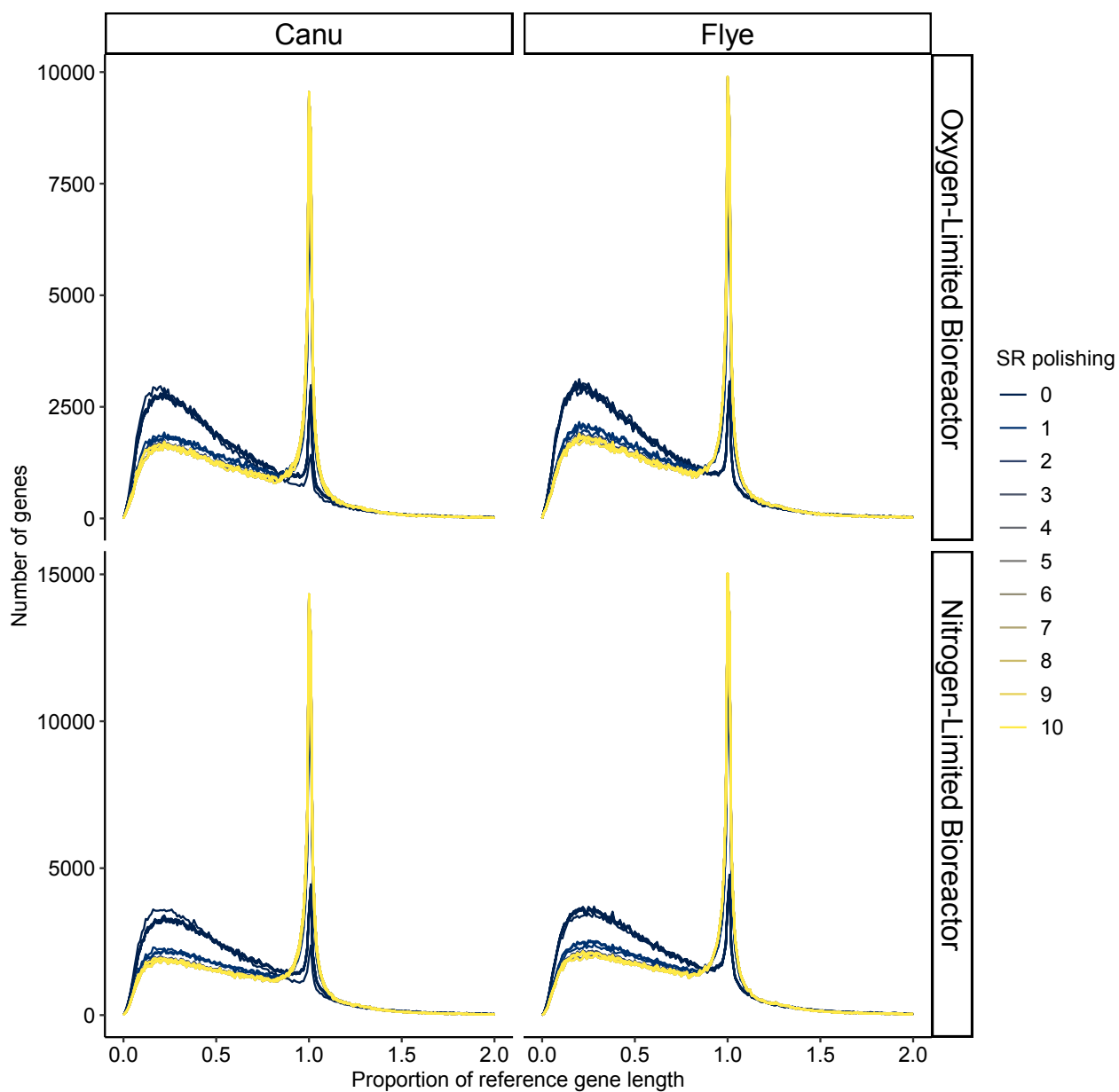

Supplement: Supplemental Information 17 — The two bioreactors are separated over vertical panels, the two LR assemblers over the horizontal panels. Colored lines indicate the gene sizes relative to their most similar reference sequence calculated by IDEEL for each assembly throughout the LR correction and SR polishing iterations. For clarity in the most relevant region, data are not shown for the proportion of reference gene lengths above two on the x-axis. [file peerj-12-18132-s017.pdf]

Proportion of genes within 5% of nearest reference length

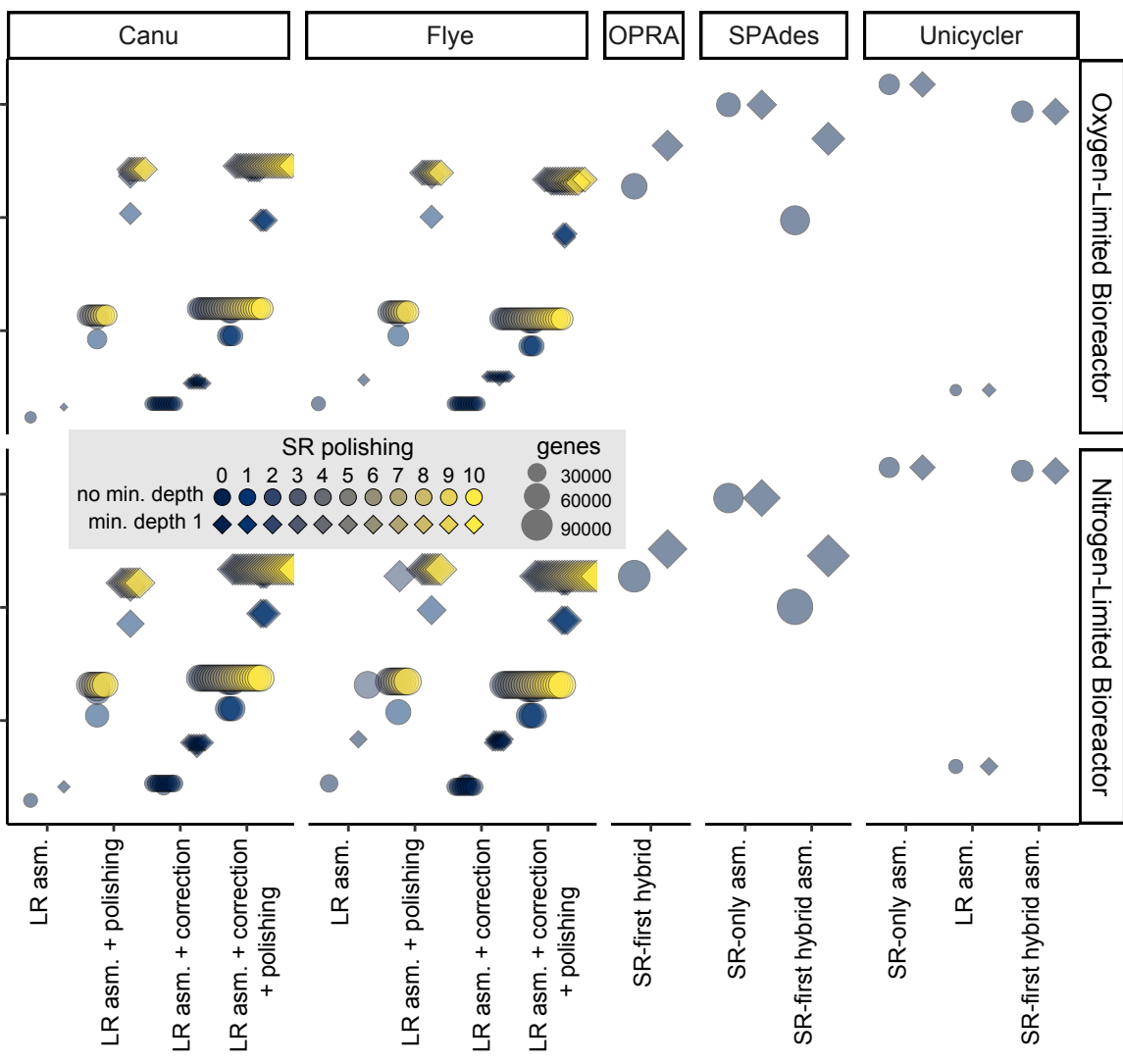

Supplement: Supplemental Information 18 — The two bioreactors are separated over vertical panels, the assemblers over the horizontal panels. Colored points represent one assembly and are grouped along the x-axis by the type of reads used for assembly and post-assembly processing. [file peerj-12-18132-s018.pdf]

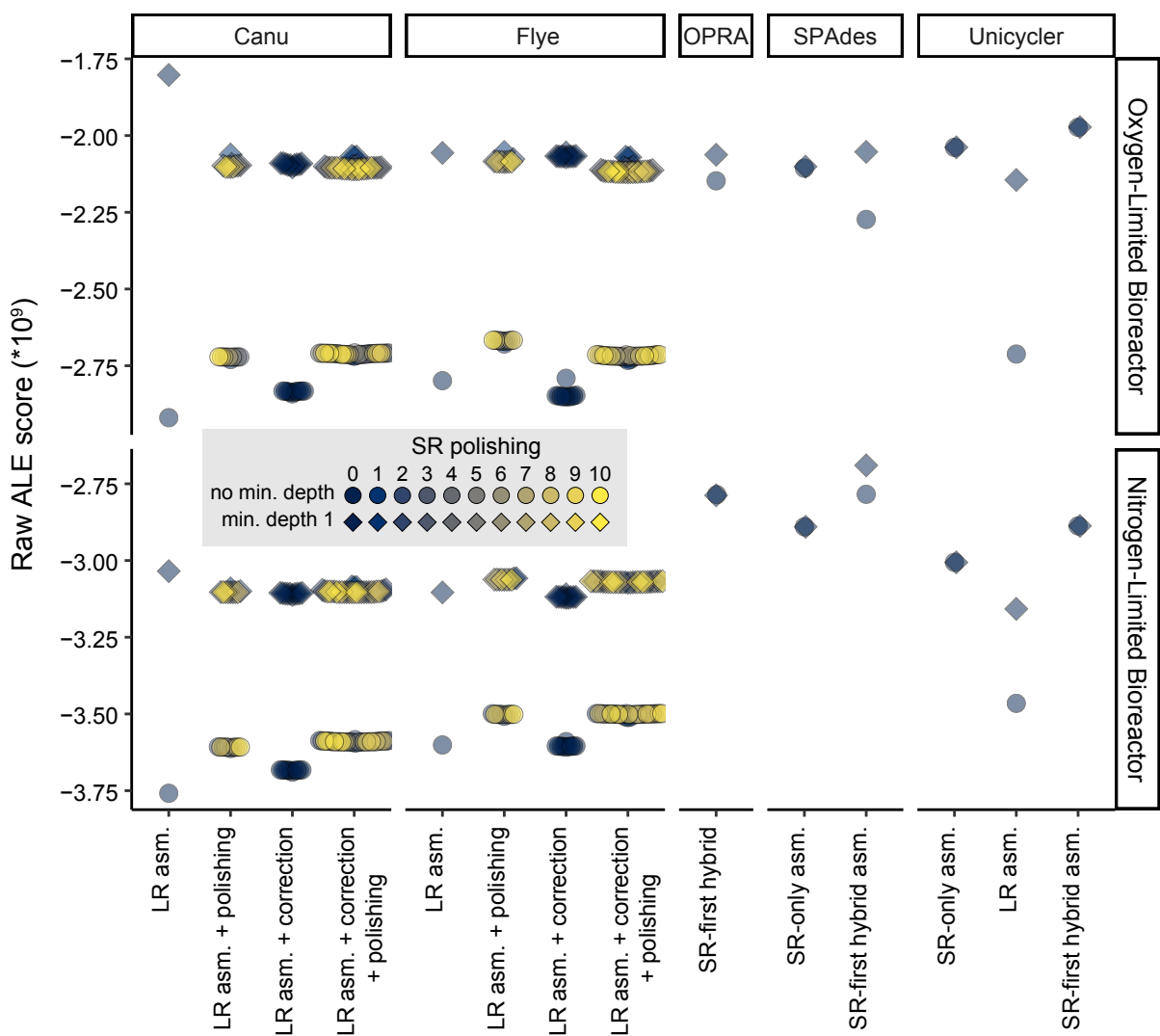

Supplement: Supplemental Information 20 — The two bioreactors are separated over the vertical panels, the assemblers over the horizontal panels. Each point represents one assembly that is colored by the SR polishing iteration, and are grouped along the x-axis by the type of reads used for assembly and post-assembly processing. [file peerj-12-18132-s020.pdf]

**A**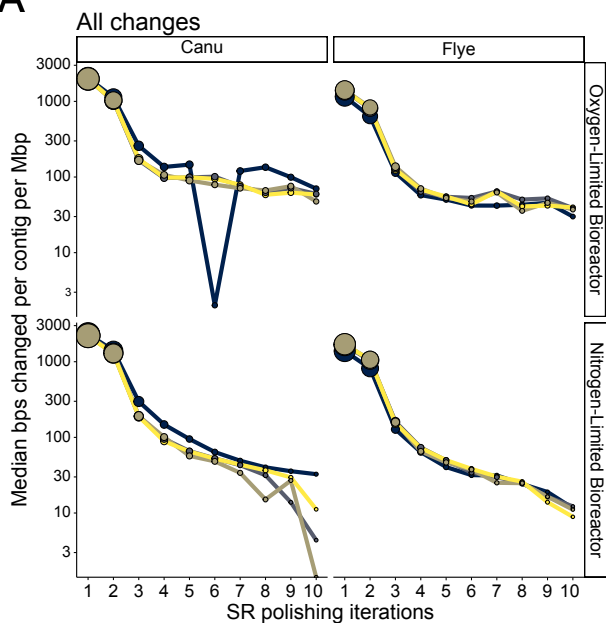

LR correction: ● 0 ● 2 ● 5 ● 10

Total number of changes per contig per Mbp: ○ 100 ○ 500 ○ 1000 ○ 1500 ○ 2000 ○ 2500

**B**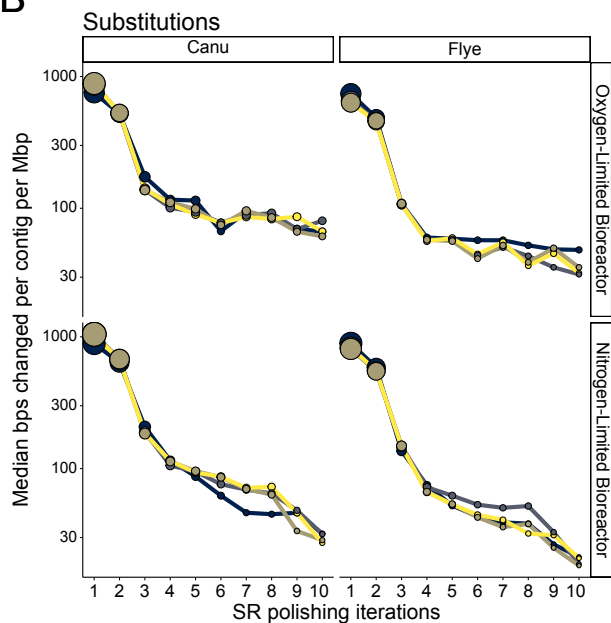**C**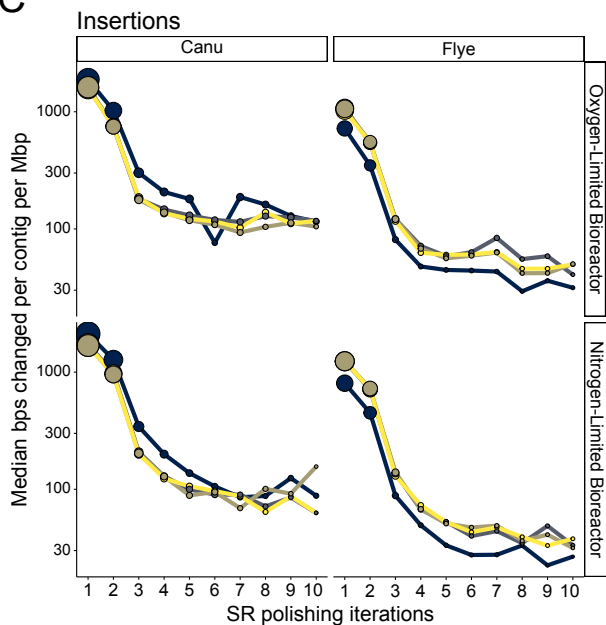**D**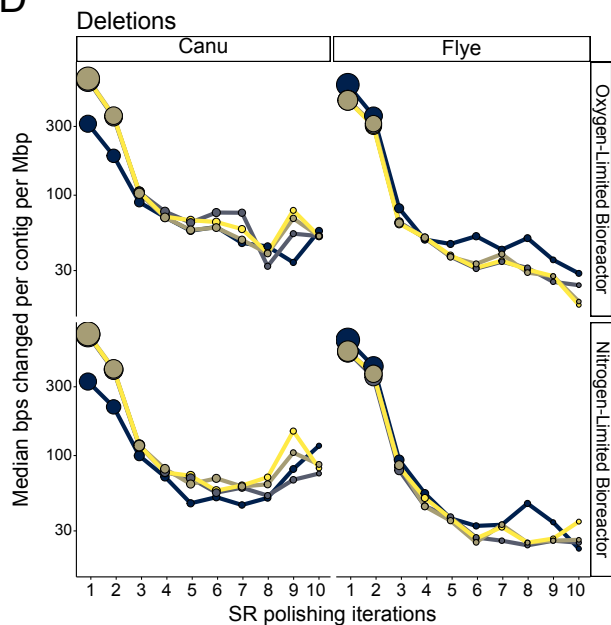

Supplement: Supplemental Information 21 — (A) All changes made during SR polishing. (B) Substitutions made during SR polishing. (C) Insertions made during SR polishing. (D) Deletions made during SR polishing. Within each main panel the two bioreactors are separated over vertical sub-panels, and the two LR assemblers are separated over the horizontal sub-panels. The x-axis shows the SR polishing iterations. Points are the median changes per contig per Mbp sequence for each assembly and are area-scaled by the median total bps changed per contig per Mbp sequence. Each point is colored by the number of preceding LR correction iterations, with colored lines connecting subsequent SR polishing iterations. [file peerj-12-18132-s021.pdf]

A

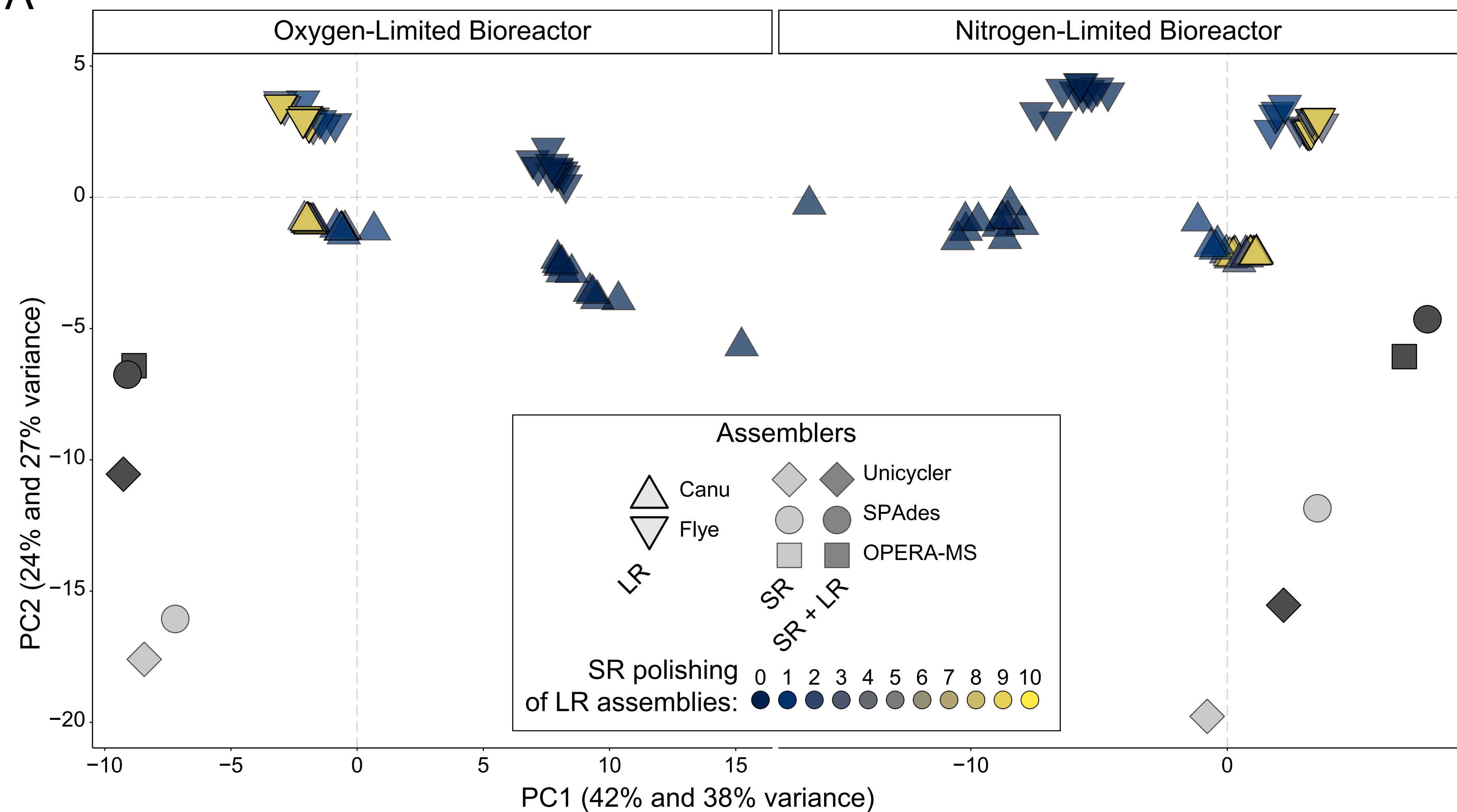

# B

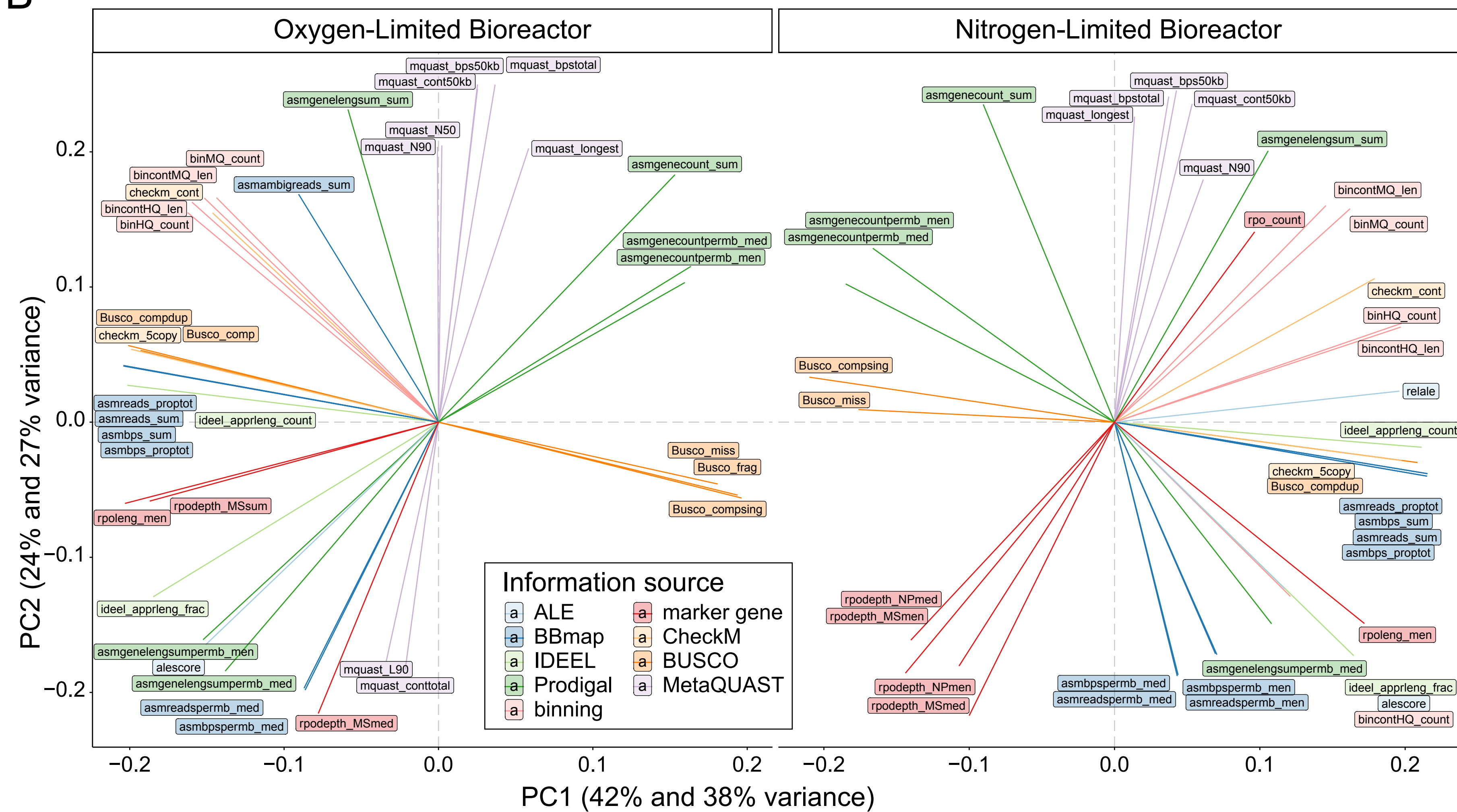

Supplement: Supplemental Information 22 — (A) Clusters of assemblies with points of different shapes indicating the assembler programs and their settings, colored by the number of short-read polishing iterations. (B) Forcings (at least 2/3 rds the max distance from 0) that structured the clusters colored by the source of the information. For both (A) and (B), bioreactors are separated over horizontal sub-panels. See Dataset S1 for a description of loadings in (B). [file peerj-12-18132-s022.pdf]
