# Supplementary material for: Simple, reference-independent assessment to empirically guide correction and polishing of hybrid microbial community metagenomic assembly
Source: PeerJ. 2024 Nov 8;12:e18132. doi: 10.7717/peerj.18132 (PMC11552494; doi:10.7717/peerj.18132)
Supplement: Supplemental Information 12 — The two bioreactors are separated over vertical panels, the two LR assemblers over the horizontal panels. The LR correction and SR polishing iterations are spread across the x-axis so that the ten SR polishing steps are immediately to the right of the preceding LR correction step. Colored bars show the CheckM copy number estimates (maximum reported is “5+”) for the entire assembly, the gray lines indicate additional information for automated bins scaled to overlay the bars for several fractions of the assemblies throughout the LR correction and SR polishing iterations. [file peerj-12-18132-s012.pdf]

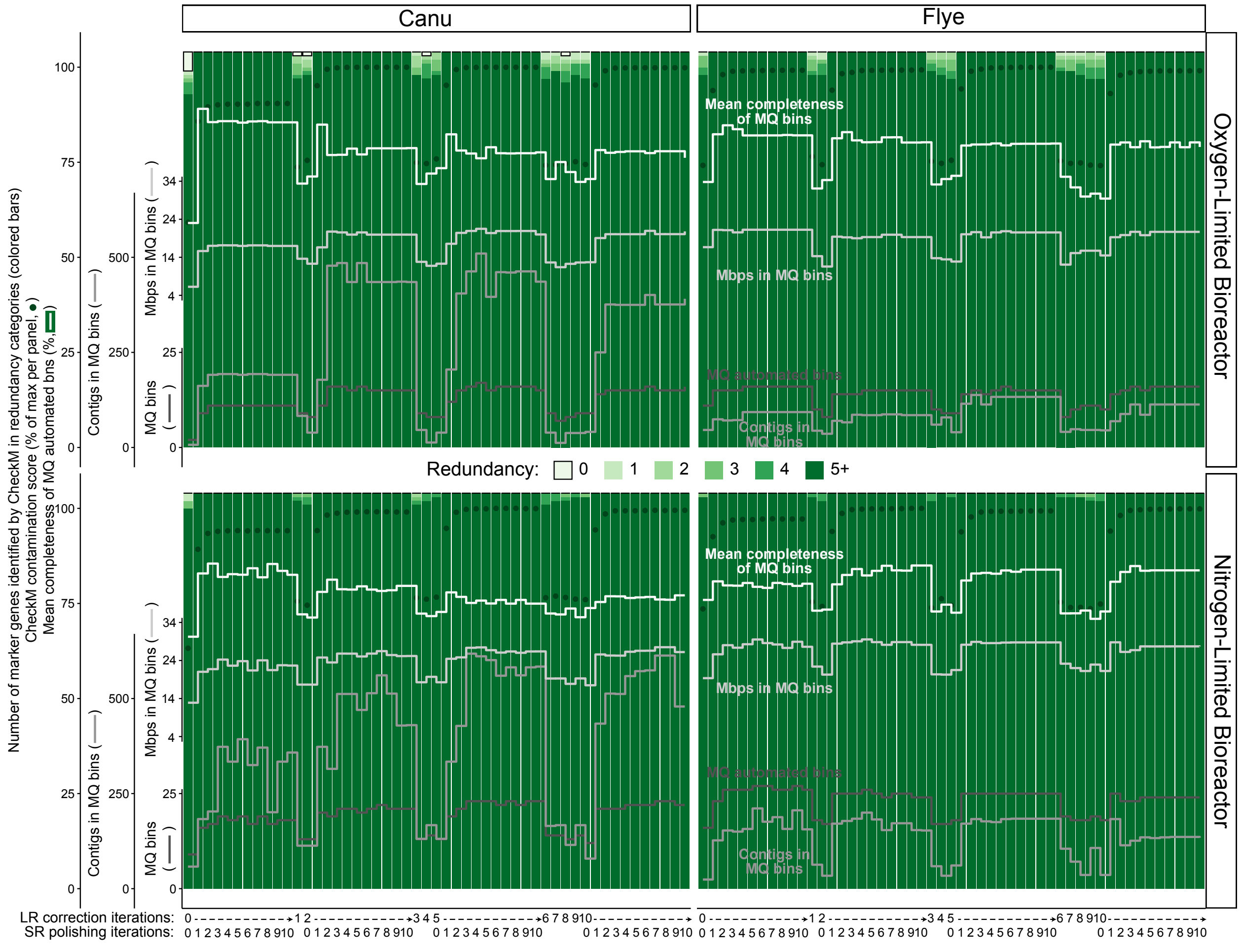

Canu
Flye
Oxygen-Limited Bioreactor
Nitrogen-Limited Bioreactor
Number of marker genes identified by CheckM in redundancy categories (colored bars)
CheckM contamination score (% of max per panel, ●)
Mean completeness of MQ automated bns (%, ▯)
Contigs in MQ bins (—)
Mbps in MQ bins (—)
MQ bins (—)
Mean completeness of MQ bins
Mbps in MQ bins
MQ automated bins
Contigs in MQ bins
Redundancy: 0 1 2 3 4 5+
LR correction iterations: 0 1 2 3 4 5 6 7 8 9 10
SR polishing iterations: 0 1 2 3 4 5 6 7 8 9 10
