# Supplementary material for: Simple, reference-independent assessment to empirically guide correction and polishing of hybrid microbial community metagenomic assembly
Source: PeerJ. 2024 Nov 8;12:e18132. doi: 10.7717/peerj.18132 (PMC11552494; doi:10.7717/peerj.18132)
Supplement: Supplemental Information 14 — The two bioreactors are separated over vertical panels, the two LR assemblers over the horizontal panels. Each point is colored by the SR polishing iteration, with grey lines connecting the points with the same number of preceding LR correction iterations, all of which are partially transparent. The green solid lines and shaded regions are the linear regressions for the displayed data and its 95% confidence interval. The broken gray lines show a slope of 1/100, representing the relationship that a redundancy score of 100 is theoretically equivalent to 1 MQ automated bin. Correlation coefficients (adjusted R2) and p-values are displayed in the upper-left corner of each panel. [file peerj-12-18132-s014.pdf]

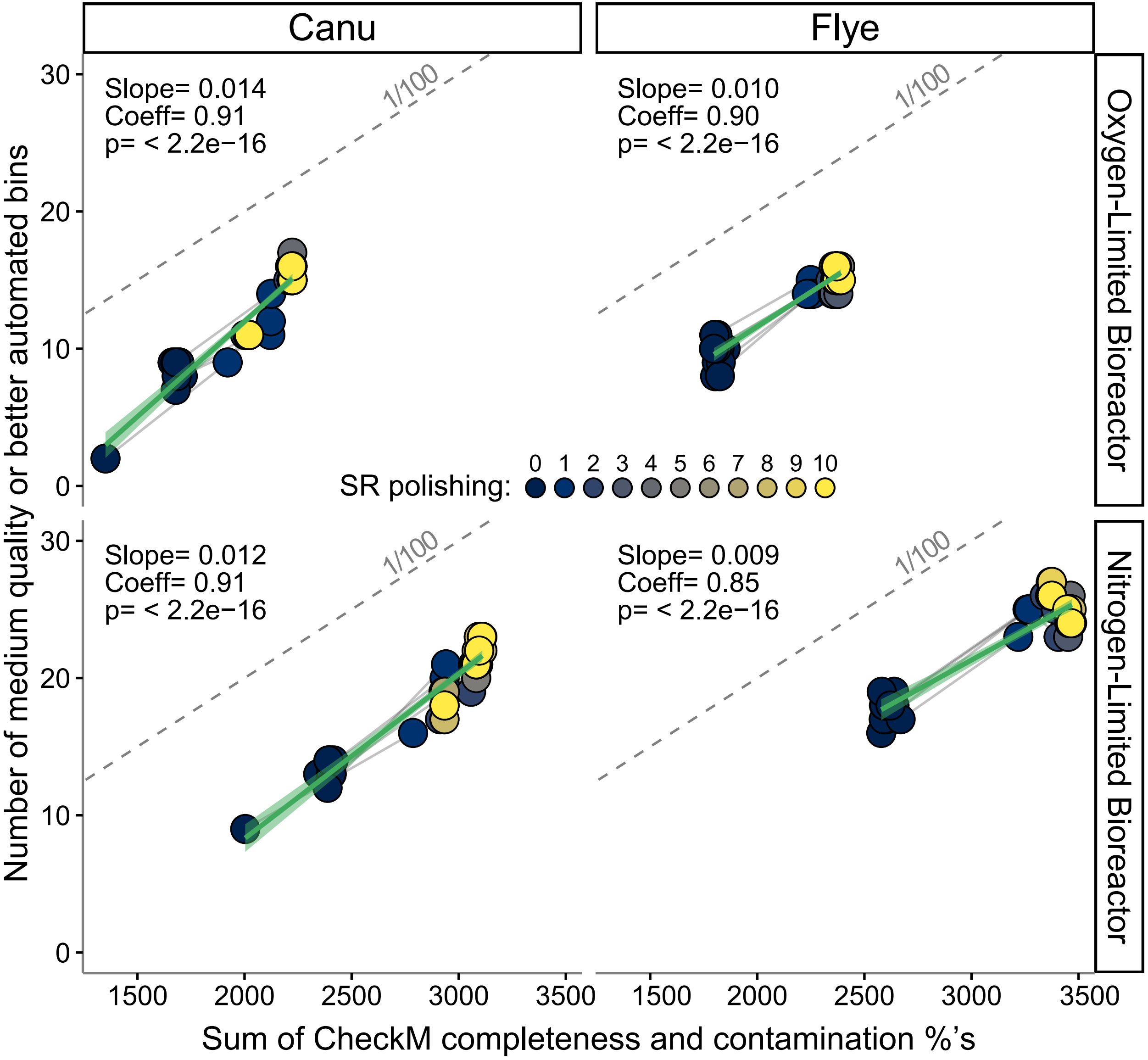
Canu
Flye
Oxygen-Limited Bioreactor
Nitrogen-Limited Bioreactor
Slope= 0.014
Coeff= 0.91
p= < 2.2e−16
Slope= 0.010
Coeff= 0.90
p= < 2.2e−16
Slope= 0.012
Coeff= 0.91
p= < 2.2e−16
Slope= 0.009
Coeff= 0.85
p= < 2.2e−16
1/100
SR polishing: 0 1 2 3 4 5 6 7 8 9 10
Number of medium quality or better automated bins
Sum of CheckM completeness and contamination %'s
0
10
20
30
1500
2000
2500
3000
3500
