# Supplementary material for: Simple, reference-independent assessment to empirically guide correction and polishing of hybrid microbial community metagenomic assembly
Source: PeerJ. 2024 Nov 8;12:e18132. doi: 10.7717/peerj.18132 (PMC11552494; doi:10.7717/peerj.18132)
Supplement: Supplemental Information 19 — The two bioreactors are separated over vertical panels, the two LR assemblers over the horizontal panels. Each point is colored by the SR polishing iteration, with grey lines connecting the points with the same number of preceding LR correction iterations, all of which are partially transparent. The purple solid lines and shaded regions are the linear regressions for the displayed data and its 95% confidence interval. Correlation coefficients (adjusted R2) and p-values are displayed in the upper-left corner of each panel. [file peerj-12-18132-s019.pdf]

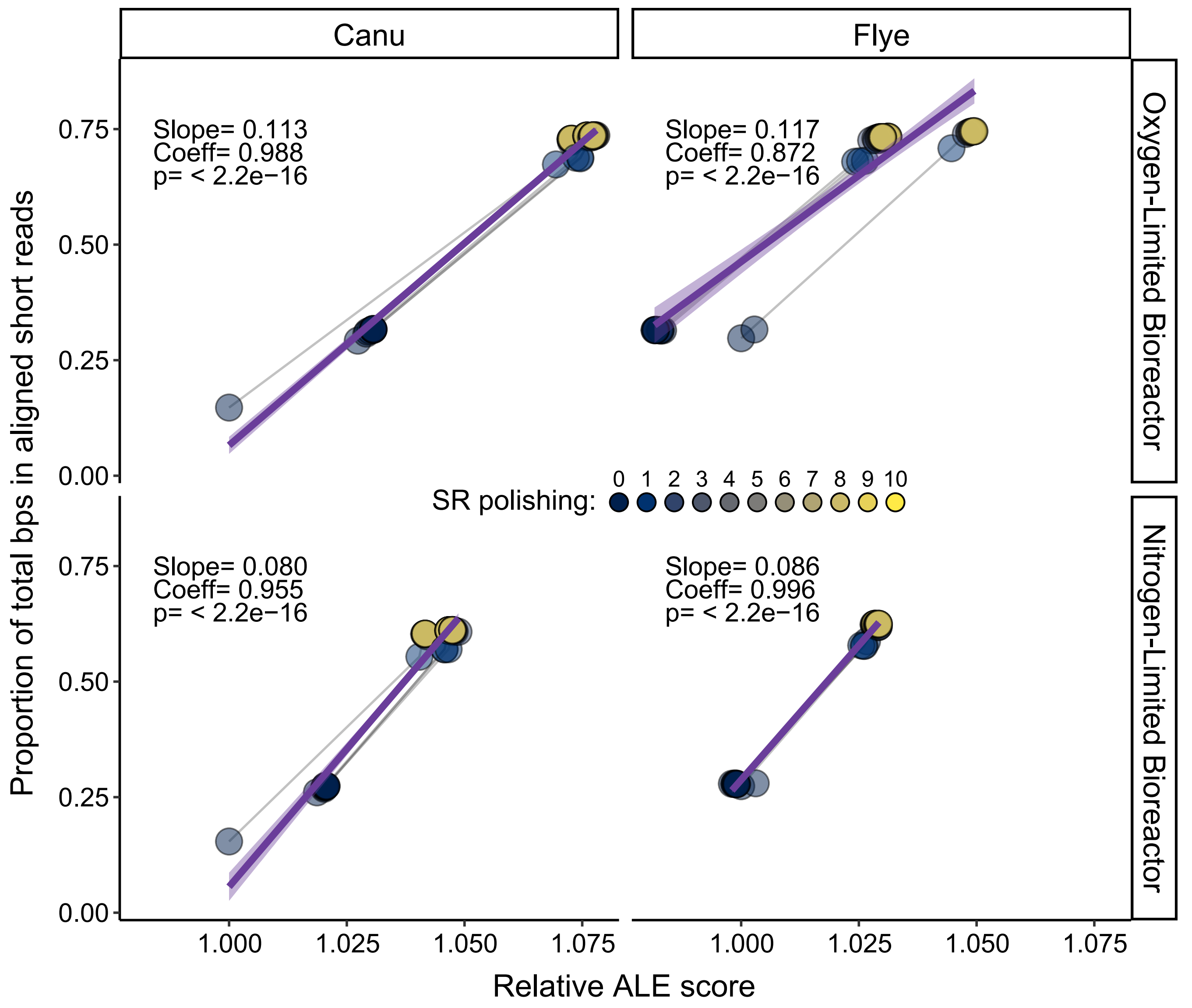
Canu
Flye
Oxygen-Limited Bioreactor
Nitrogen-Limited Bioreactor
Slope= 0.113
Coeff= 0.988
p= < 2.2e−16
Slope= 0.117
Coeff= 0.872
p= < 2.2e−16
Slope= 0.080
Coeff= 0.955
p= < 2.2e−16
Slope= 0.086
Coeff= 0.996
p= < 2.2e−16
SR polishing: 0 1 2 3 4 5 6 7 8 9 10
0.00
0.25
0.50
0.75
1.000
1.025
1.050
1.075
Proportion of total bps in aligned short reads
Relative ALE score
